# Acceptability y feasibility of “Yo Sé Lo Que Quiero (YSLQQ)” (Unplugged) program: a drug prevention intervention for adolescents in Chile

**DOI:** 10.1192/j.eurpsy.2022.624

**Published:** 2022-09-01

**Authors:** G. Salgado, S. Ramirez, S. Gana, M. Valenzuela, J. Gaete

**Affiliations:** 1 Universidad de los Andes, Epidemiology And Health Studies, Santiago, Chile; 2 Universidad de los Andes, Faculty Of Education, Santiago, Chile

**Keywords:** prevention, Adolescents, substance use, Acceptability

## Abstract

**Introduction:**

Substance misuse among adolescents is a public health problem because of its prevalence and consequences.

**Objectives:**

i) To develop a culturally adapted version of the Unplugged program in Chile, renamed as “Yo Sé Lo Que Quiero (YSLQQ)”, for substance use prevention; ii) To evaluate the acceptability and feasibility of its implementation.

**Methods:**

Pilot study, with randomized controlled trial design, with two arms (Intervention Group, IG; and Control Group, CG). The sample consisted of 1,556 students from 6th to 8th grade from six schools (1:1 ratio) in Santiago, Chile. The IG received the 12 sessions of the YSLQQ program and the CG the usual substance use prevention activities. Acceptability was assessed through a student questionnaire, and feasibility through teacher self-report.

**Results:**

More than half of the students reported that they liked the sessions. 61.3% were satisfied with the duration of the program and 61.7% with the activities. 68% of students agreed that the program helped them to have more refusal skills towards tobacco, alcohol, and drug use in the future. On the other hand, concerning feasibility and fidelity, 88.9% of the teachers remained faithful to the manual, and 91.6% of the activities were fulfilled according to the manual.

**Conclusions:**

The present study demonstrated that “Yo Sé Lo Que Quiero” program is acceptable and feasible for future implementation in adolescents.

**Disclosure:**

No significant relationships.

